# Metabolomics approach to serum biomarker for laxative effects of red *Liriope platyphylla* in loperamide-induced constipation of SD rats

**DOI:** 10.1186/s42826-019-0009-x

**Published:** 2019-07-24

**Authors:** Ji Eun Kim, Young Ju Lee, Sung Ha Ryu, Ji Won Park, Mi Ju Kang, Hyeon Jun Choi, Su Ji Bae, Yusang Choi, Hyun Gu Kang, Kyu-Bong Kim, Suhkmann Kim, Yong Lim, Dae Youn Hwang

**Affiliations:** 1 Department of Biomaterials Science, College of Natural Resources & Life Science / Industry Convergence Research Institute, Pusan National University, 50 Cheonghak-ri, Samnangjin-eup Miryang-si, Gyeongsangnam-do 627-706 South Korea; 2 Analysis Research Team, R&D Center, GL Pharm Tech Corp, Gyeonggi-do, 13202 Republic of Korea; 3 Laboratory of Veterinary Theriogenology, Department of Veterinary Medicine, College of Veterinary Medicine, Chungbuk National University, Cheongju, 28644 South Korea; 4 College of Pharmacy, Dankook University, Chungnam, 330-714 South Korea; 5 Department of Chemistry and Chemistry Institute for Functional Materials, Pusan National University, Busan, 609-735 South Korea; 6 Department of Clinical Laboratory Science, College of Nursing and Healthcare Science, Dong-Eui University, Busan, South Korea

**Keywords:** Red *Liriope platyphylla*, Constipation, Laxative effects, Metabolomics, Serum

## Abstract

Red *Liriope platyphylla* (RLP) is a known herbal medicine used in the treatment of some chronic diseases including constipation, neurodegenerative disorders, diabetes and obesity. To determine and characterize putative biomarkers that predict the laxative effects induced by RLP treatment, alteration of endogenous metabolites was measured in the serum of loperamide (Lop)-induced constipation rats after administration of RLP extract (EtRLP) using ^1^H nuclear magnetic resonance (^1^H NMR) spectral data. The urine volume and amounts, and weights and water contents of stools were significantly recovered in the Lop + EtRLP treated group as compared to the No group, whereas body weight and food intake maintained constant levels. Also, significant recoveries in the thickness of mucosa and muscle were detected in the colon of the Lop + EtRLP treated group. Furthermore, pattern recognition showed absolutely different clustering of the serum analysis parameters when comparing the Lop treated group and Lop + EtRLP treated group. Of the 33 endogenous metabolites, 7 amino acids (alanine, arginine, glutamate, glutamine, glycine, threonine and valine) and 8 endogenous metabolites (betaine, creatine, glucose, taurine, ethanol, lactate, glycerol and succinate) were dramatically increased in the Lop + EtRLP treated SD rats. These results provide the first evidence pertaining to metabolic changes in the constipation rats treated with Lop + EtRLP. Additionally, these findings correlate with changes observed in 15 metabolites during the laxative effects of EtRLP.

**Electronic supplementary material:**

The online version of this article (10.1186/s42826-019-0009-x) contains supplementary material, which is available to authorized users.

## Introduction

Constipation is a bowel disease characterized by symptoms such as infrequent bowel movements, difficulty during defecation, and sensation of incomplete bowel evacuation [1–4]. This disease is often the outcome of numerous reasons including insufficient dietary fiber intake, inadequate fluid intake, decreased physical activity, side effects of medication, hypothyroidism, obstruction by colorectal cancer, and side effects due to drug administrations [5–7]. Several chemical drugs and natural products have been investigated as laxatives to treat constipation. Chemical drugs (laxatives) such as senna, correctol, exlax, senokot and gaviscon are widely prescribed to increase bulkiness and soften stool, or as osmotic agents that enhance water flow into the colon to promote elimination and trigger bowel movements, although they have undesirable side effects [8, 9]. Furthermore, several herbal medicines such as *Aloe ferox* Mill., *Liriope platyphylla*, *Fumaria parviflora* and *Mareya micrantha* Mull are known to dramatically improve the symptoms of constipation in animal models, without any significant adverse side effects [6, 10, 11]. In an effort to identify novel drugs for the treatment of constipation, a recent study has investigated the therapeutic effects of Red *Liriope platyphylla* extract (EtRLP) in Lop-induced constipated SD rats [12].

EtRLP was manufactured from *L. platyphylla* roots using a specific process consisting of two steps (steaming of *L. platyphylla* roots at 99 °C for 3 h, followed by air-drying at 70 °C for 24 h) for different number of repetitions [13]. The extract contains a large amount of total phenolic compounds, total flavonoids and 5-hydroxmethyl-2-furfural, and is mainly composed of carbohydrates, moisture, crude protein, crude ash, and crude fat [5]. Furthermore, EtRLP treatment induces the enhancement of insulin secretion and decrease in the glucose concentration in INS cells, STZ-induced diabetic ICR mice, and OLETF rats [5, 13, 14]. Furthermore, the symptoms of constipation, including stool excretions, histological structure, mucin section, muscarinic acetylcholine receptors (mAChR) signaling pathway, endoplasmic reticulum (ER) stress response, and mucin secretion are markedly improved after the RLP administration [12]. However, to date, there has been no research in screening of metabolomics biomarkers that anticipate the laxative effects induced by RLP treatment.

As part of the search for sensitive and reliable biomarkers of laxative effects, this study was designed to compare the serum biomarkers obtained from Lop + EtRLP treated SD rats using the metabolomics-based proton (NMR) platform. The present results indicate that the metabolomics profile of serum collected from EtRLP treated constipation SD rats may provide useful information in the future development of novel sensitive and reliable biomarkers for laxative drugs.

## Results

### Effect of EtRLP on the feeding behavior and excretion parameters of the constipation rats

**Fig. 1:**
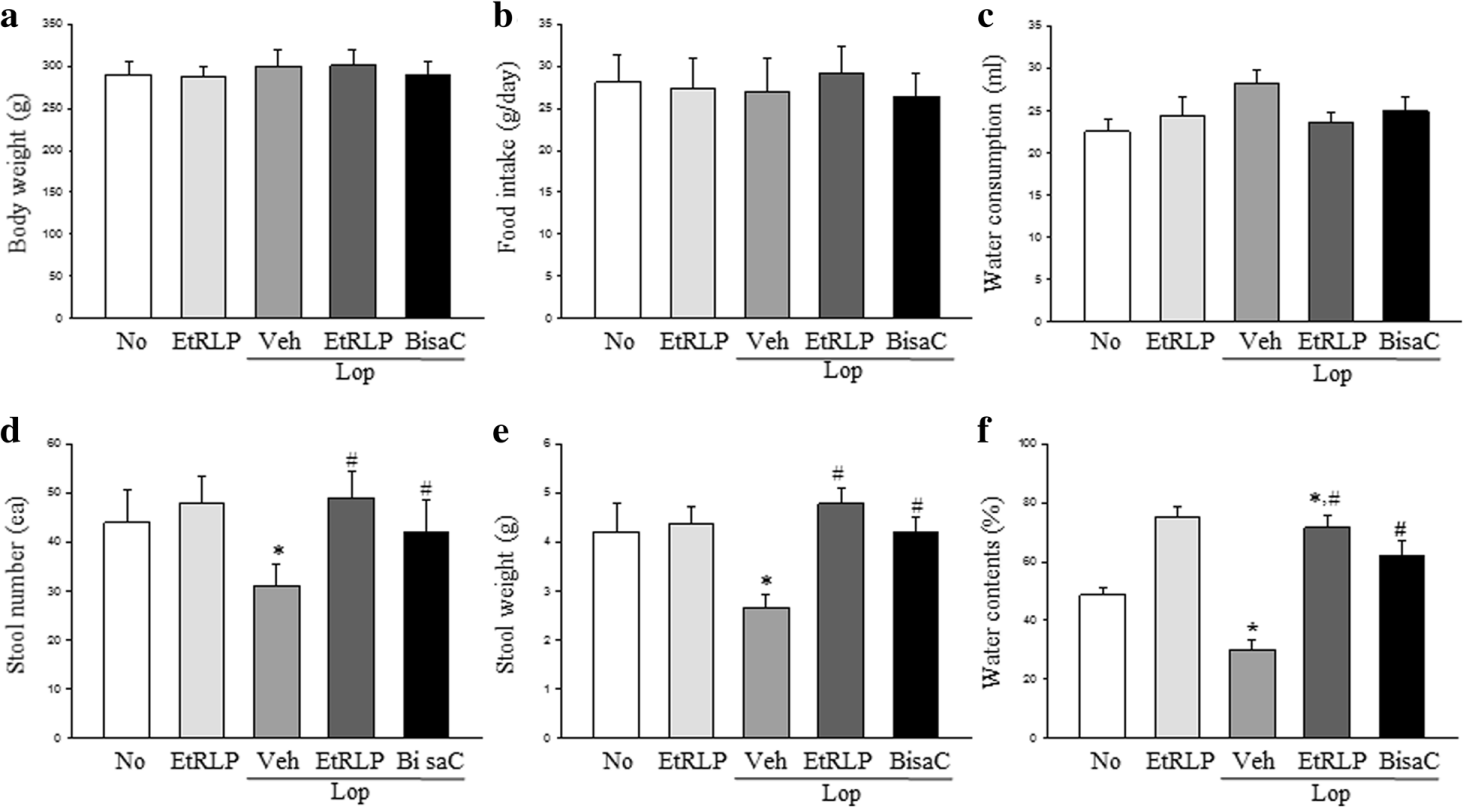
Feeding behavior and stool parameters after EtRLP treatment. **a**-**c** Three parameters related with the feeding behavior were measured in SD rats of subset groups in duplicate. **d**-**e** Three stool parameters were measured in SD rats of subset groups in duplicate. Five to six rats per group were assayed in duplicate by parameter counting. The data are reported as the mean ± SD. * indicates *p* < 0.05 compared to the NO group. # indicates *p* < 0.05 compared to the Lop + Veh treated group

To investigate whether EtRLP treatment affects the feeding behavior and excretion parameters of constipated rats, we measured the food intake, water consumption and excretion parameters in SD rats of subset groups. As shown in Fig. 1, water consumption was slightly recovered in the Lop + EtRLP treated group, whereas no significant difference was observed in the body weight and food intake of the LOP treated and Lop + EtRLP treated groups. Also, a decrease in stool number, weight and water contents in the LOP treated group was almost recovered in the Lop + EtRLP and LOP + BisaC treated groups. These results indicate that EtRLP treatment improves the excretion parameters of Lop-induced constipation in SD rats without any changes in the feeding behavior.

### Recovery effect of EtRLP on the histological alteration of colon

**Fig. 2:**
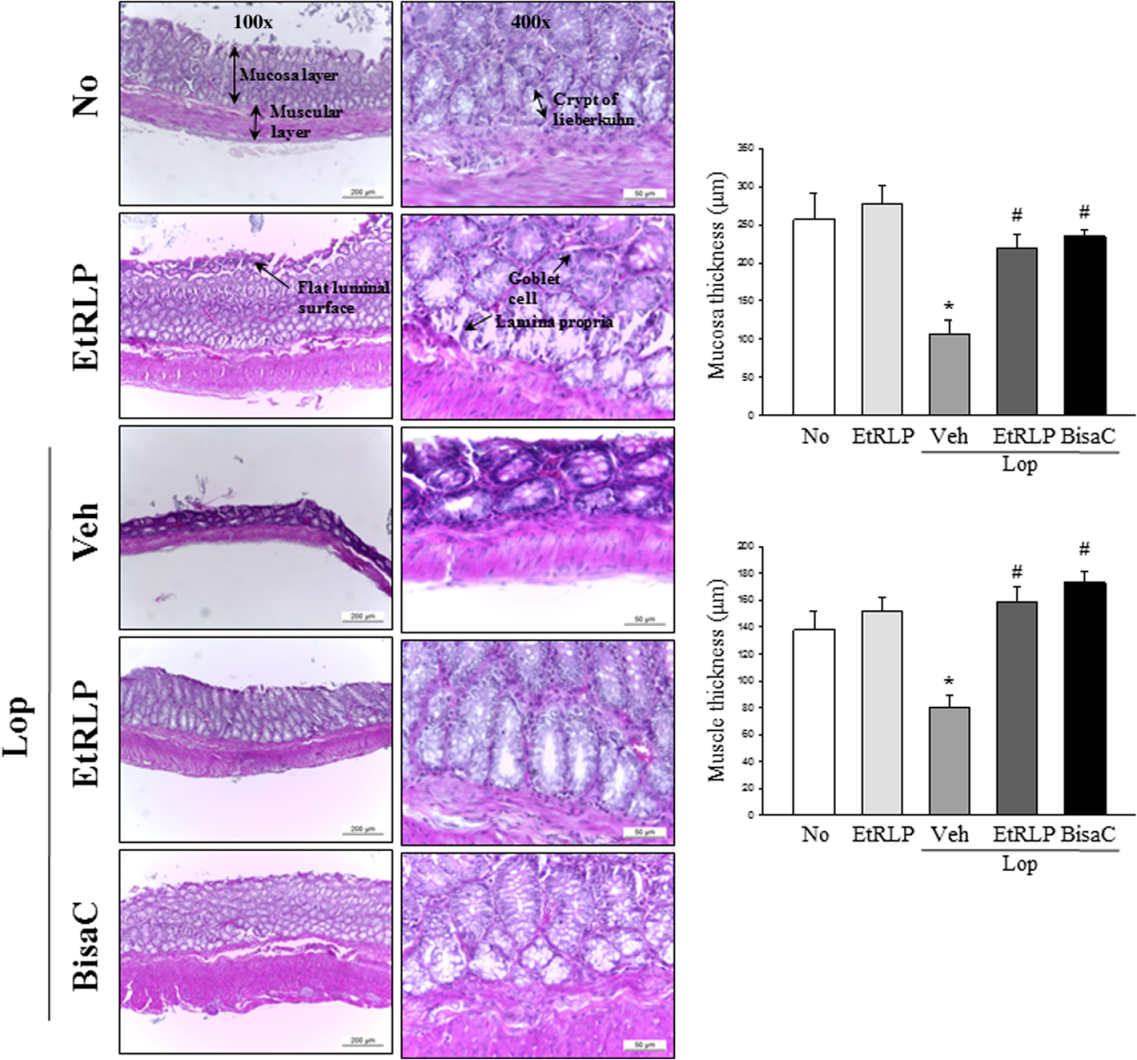
Histological structure of EtRLP treated colon. H&E-stained sections of transverse colons collected from No, EtRLP, Lop + Vehicle, Lop + EtRLP and Lop + BisaC treated rats were observed at two different magnifications (100x and 400x) using a light microscope. Five to six rats per group were assayed in triplicate, after H&E staining. The data are reported as the mean ± SD. * indicates p < 0.05 compared to the NO group. # indicates *p* < 0.05 compared to the Lop + Veh treated group

To investigate the effects of EtRLP treatment on the recovery of the histological structure of the colon, we evaluated alterations in mucosa and muscle thickness in colon sections of subset groups stained with H&E. The average thickness of mucosa and muscle was observed to be significantly shorter in the Lop + Veh treated group as compared to the No treated group. Following Lop + EtRLP or LOP + BisaC cotreatment, this level greatly increased when compared with the Lop + Vehicle treated group (Fig. 2). These results suggest that EtRLP induces in recovering the abnormal structure of the colon in constipated SD rats after Lop treatment.

### Effects of EtRLP treatment on endogenous metabolites in serum

**Fig. 3:**
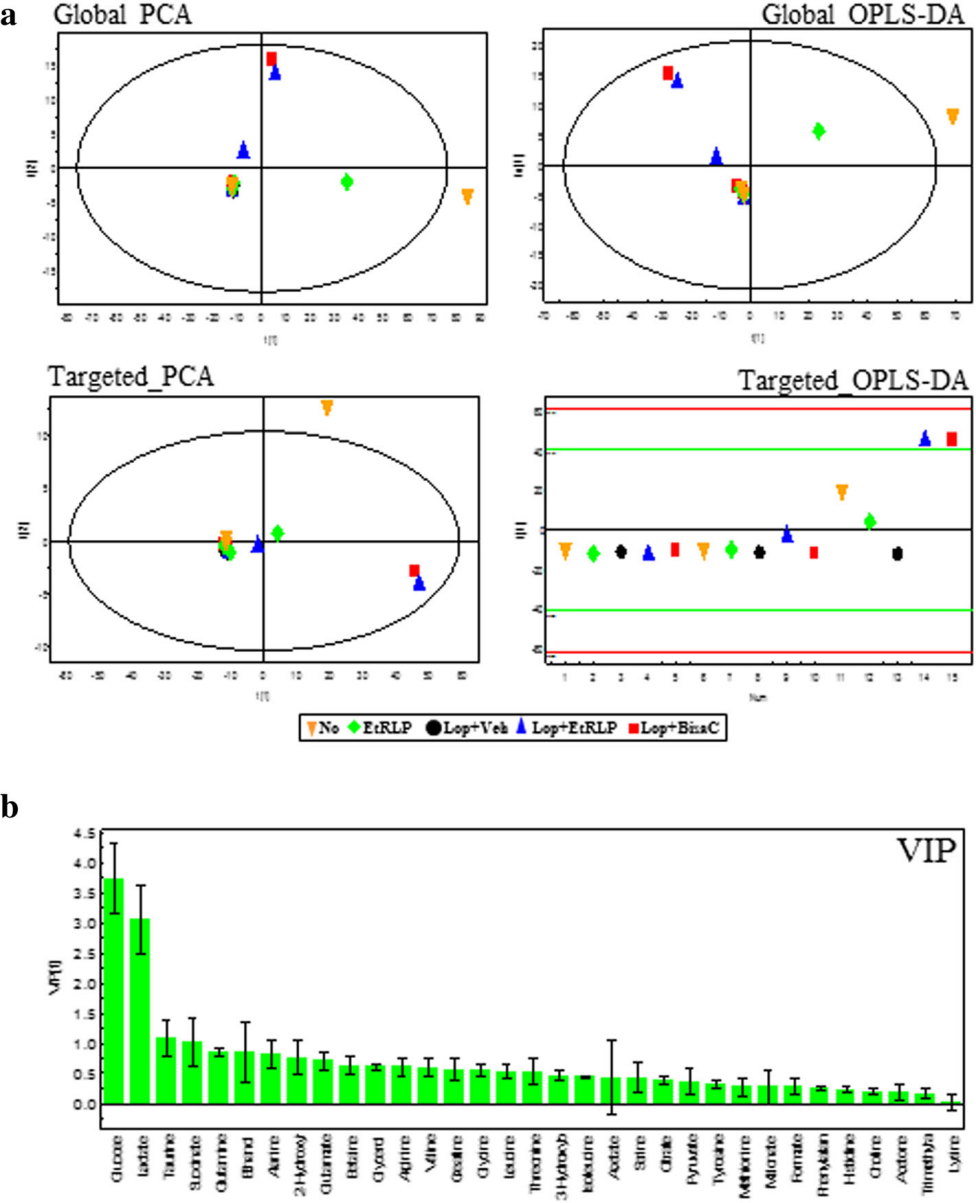
Spectral analysis of metabolomic pattern using PCA and OPLS-DA. **a** Global profiling of loperamide treatment in serum samples. Targeted profiling of loperamide treatment in serum samples. **b** The VIP shows the major metabolites contributing to cluster separation. The data are reported as the mean ± SD

**Fig. 4:**
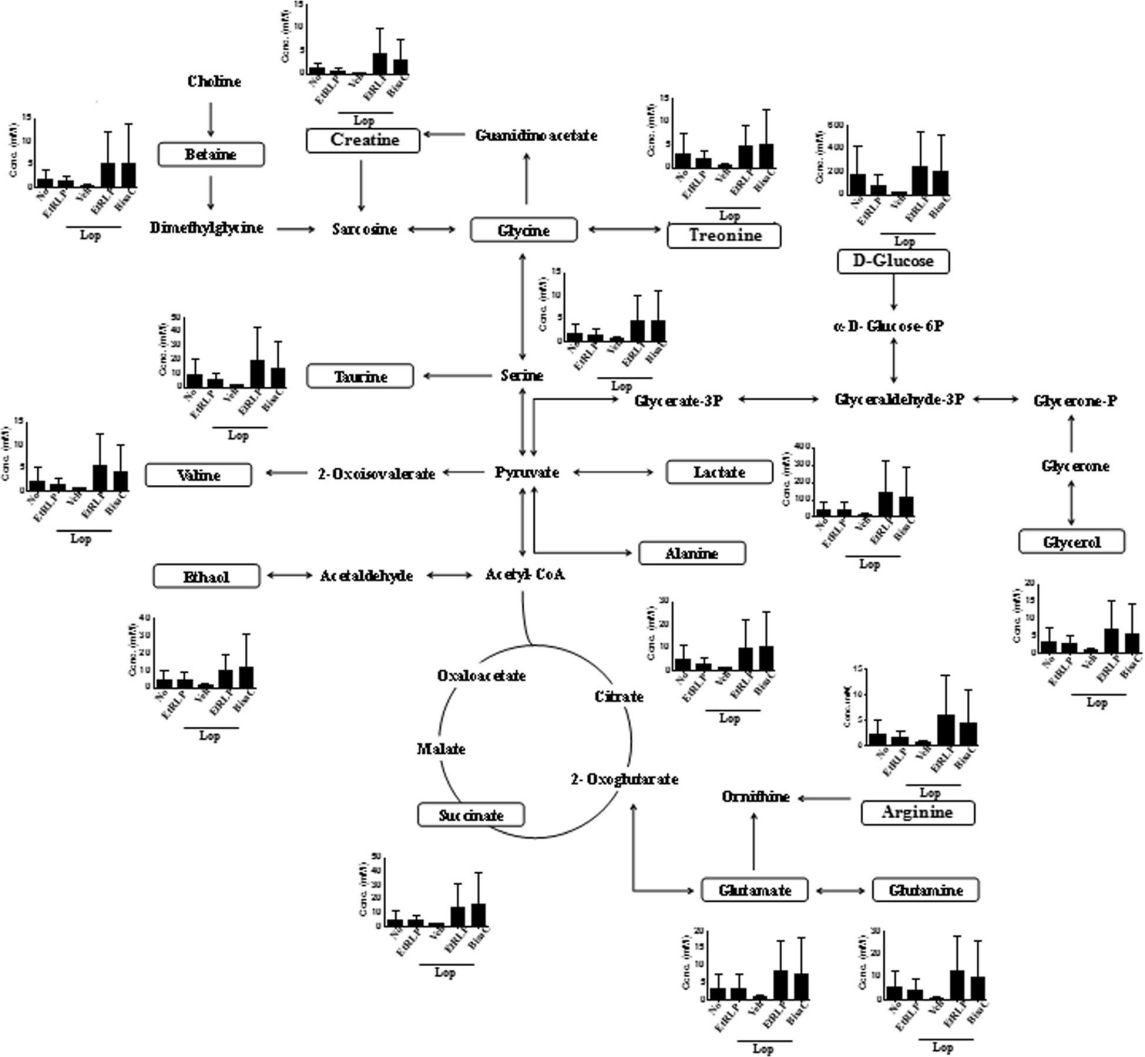
Concentration of 7 amino acids and 8 metabolites after EtRLP administration in SD rats. The pathway relates to the endogenous metabolites that are altered after Lop + EtRLP treatment, while the box shows the altered metabolites. The data are reported as the mean ± SD

To observe the effects of EtRLP treatment on the endogenous metabolites of subset groups, pattern recognitions were analyzed in the serum of subset groups using PCA and OPLS-DA. NMR spectra in global profiling show clustering between the No group and Lop + Veh treated group, or between Lop + Veh treated group and Lop + EtRLP treated group (Fig. 3a). Also, the Chenomx NMR Suite (ver. 4.6, Chenomx Inc., Edmonton, Alberta, Canada), applied for targeted NMR spectral analysis, detected a total of 33 metabolites (Additional file 1: Table S1); the PCA and OPLS-DA score plots differed between the No- and Lop + Veh treated groups (Fig. 3b). Of these, some endogenous metabolites were selected to investigate the effects of EtRLP treatment using VIP. Especially, 15 metabolites were detected in response to EtRLP treatment. These metabolites were categorized into two major groups: an amino acid group containing alanine, arginine, glutamine, glutamate, glycine, threonine and valine, and a metabolite group containing betaine, creatine, glycerol, glucose, ethanol, lactate, succinate and taurine. In serum samples of the Lop + Veh treated group, these metabolite concentrations were significantly decreased relative to those of the No treated group. However, significant recovery was observed after Lop + EtRLP cotreatment. Furthermore, the level of most metabolites in the Lop + EtRLP treated group was very similar with those of the Lop + BisaC treated group (Fig. 4). Therefore, these results suggest that EtRLP treatment causes a recovery of 15 endogenous metabolites relative to the constipations.

### Confirmation of glucose and creatine concentration

**Fig. 5:**
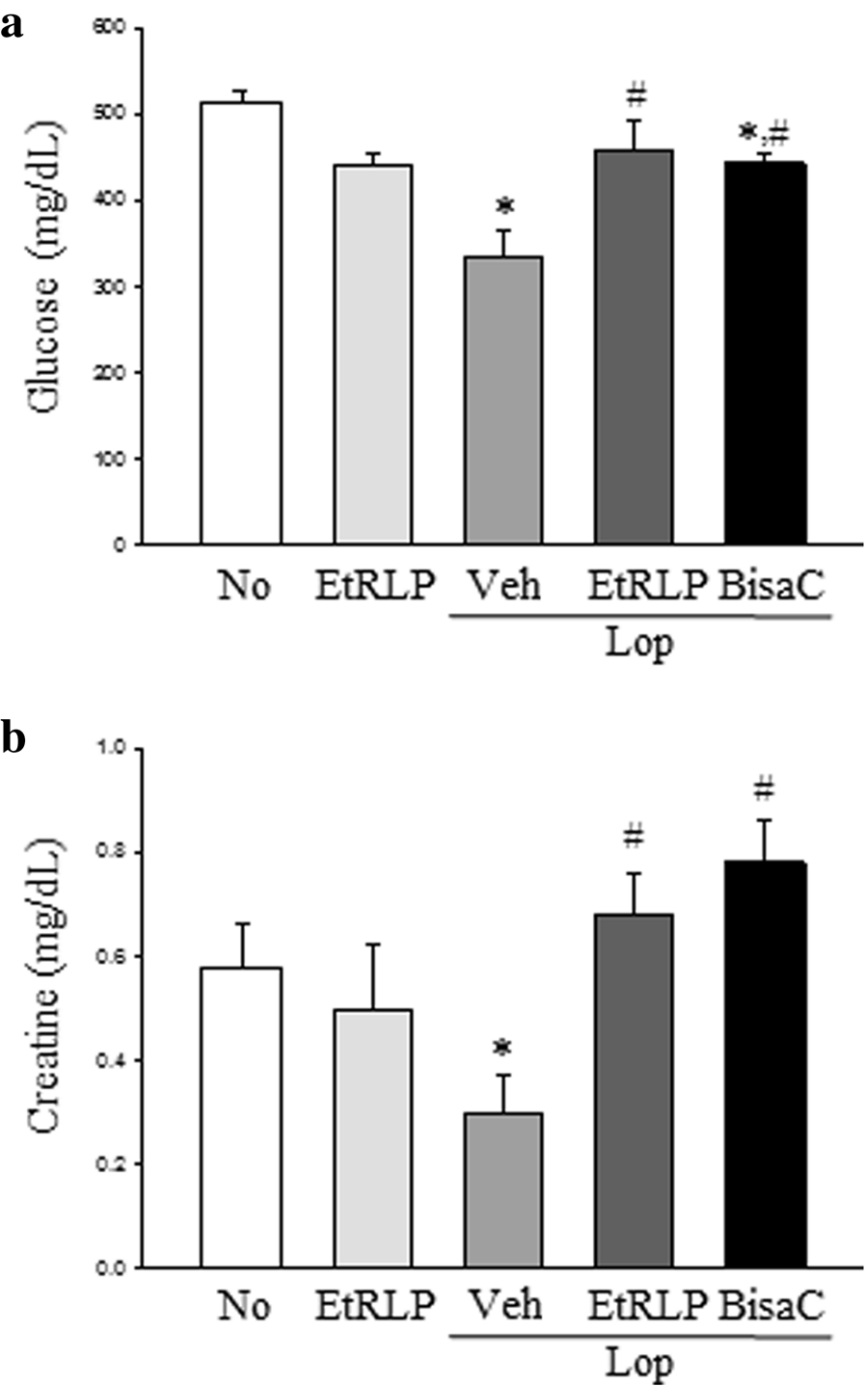
Serum biochemical analysis for glucose **a** and creatine **b** concentration. After the collection of serum, their levels were measured using an automatic biochemical analyzer. Five to six rats per group were assayed in duplicate by parameter counting. The data are reported as the mean ± SD. * indicates *p* < 0.05 compared to the NO group. # indicates *p* < 0.05 compared to the Lop + Veh treated group

Serum biochemical analyses were conducted to validate the changes in the concentration of the two metabolites identified by metabolomics analysis. As shown in Fig. 5, the regulation pattern of glucose and creatine observed upon serum biochemical analyses was very similar to those in NMR spectrometry. They were significantly enhanced in Lop + EtRLP or Lop + BisaC treated group, although their levels were significantly lower in the Lop + Veh treated group than No treated group. Therefore, these results indicate that the alteration of metabolites detected by NMR spectrometry reflects change in metabolite concentration in the serum of the Lop + EtRLP and Lop + Veh treated group.

## Discussion

Some extracts derived from natural products are considered as key candidates for treating chronic constipation, since they are able to increase the intestinal motility, frequency and weight of stools, and the ileum tension. Especially, the extract of *Aloe ferox* Mill., agarwood (*Aquilaria sinensis* and *Aquilaria crasna*) leaves and *Ficus carica* paste induces an improvement in the intestinal motility, increases fecal volume, and normalizes the body weight in Lop-induced constipated rats [5, 11, 15]. Also, extracts of LP are reported to increase the frequency and weight of stools, villus length, crypt layer, muscle thickness, mucin secretion, and accumulation of lipid droplets, and dramatically reduce the mAChRs signal pathway, in enterocytes of the crypt in Lop-induced SD rats [10]. However, no studies have investigated alterations in the metabolomics profiles during the process of EtRLP therapeutic outcome against constipation. We believe that our study therefore provides the first evidence for metabolomics profiles obtained from the laxative effects of EtRLP in Lop-induced constipation SD rats.

Twenty amino acids are used as the major units to structure proteins and other biomolecules, and are known to oxidize to urea and carbon dioxide as key sources of energy [16]. Of these, 9 amino acid (histidine, isoleucine, leucine, lysine, methionine, phenylalanine, threonine, tryptophan and valine) are assigned as essential amino acids since they cannot be synthesized from other compounds at the concentration required for normal growth in the human body [17]. Especially, dietary amino acids are used as key substrates for the synthesis of intestinal proteins, NO and polyamines, as well as consumed as the major fuel in the small intestine [18]. Some specific amino acids, including glutamine, glutamate, arginine, glycine, lysine, threonine, and sulfur-containing amino acids, have a potentially therapeutic role against gut-related diseases [19, 20]. However, scientific evidence correlating the laxative effect of EtRLP and amino acids has not been presented to date. In the present study, the results of metabolomics profile analysis indicate that the serum level of 7 amino acids can be recovered by EtRLP treatment. Our findings also show that these amino acids may be key markers indicating the laxative effects in Lop induced constipation rats.

Meanwhile, serum has been used as an important specimen for studying the action mechanism and evaluating therapeutic efficacy in constipation and irritable bowel syndrome (IBS). The production levels of gastrointestinal metabolic components including motilin (MTL), gastrin (Gas), endothelin (ET), substance P (SP), acetylcholinesterase (AChE) and vasoactive intestinal peptide (VIP) were measured in serum to investigate the laxative effects of *Lactobacillus fermentum* Suo and naringenin [21, 22]. Serum serotonin and glucose-like peptide (GLP-1) concentration were measured by ELISA in patient with IBS [23, 24]. Serum proteomic analysis was performed upon patients with constipation predominant IBS symptoms. Among 1,317 proteins, 12 proteins, such as TGFβ1, PF4V1, PF4, APP and MMP showed significance different in level of expression [25]. Based on above previous results, we selected serum as a sample for analyzing metabolites.

The metabolic profiles associated with constipation were investigated in few studies using samples of animals and human. The metabolic profile before and after a meal challenge in a cohort of children with constipation was investigated to determine its relationship with postprandial colon motility patterns and identify metabolic targets for treatment of constipation. Some significant alterations were measured on the 16 amino acid and 22 lipid metabolites of the postprandial group among 187 metabolites. Some correlation between normal and abnormal postprandial motility pattern was detected in the concentration of specific metabolites including glycerol, carnosine, alanine, asparagine, cytosine, choline, phosphocholine, thyroxine and triiodothyronine [26]. Also, metabolomics approach was performed in the serum of Lop-induced constipation in SD rats. Among 35 endogenous metabolites, four amino acids (alanine, glutamate, glutamine and glycine) and six endogenous metabolites (acetate, glucose, glycerol, lactate, succinate and taurine) were significantly decreased in Lop-treated SD rats [27]. In this study, the Lop + EtRLP treated SD rats showed a dramatic alteration on the 7 amino acids (alanine, arginine, glutamate, glutamine, glycine, threonine and valine) and 8 endogenous metabolites (betaine, creatine, glucose, taurine, ethanol, lactate, glycerol and succinate). These patterns were very similar with metabolomics profile of Lop-induced constipation rats although they were different from the results analyzed from children with constipation. Especially, glycerol was commonly detected as an important key marker for constipation among various metabolites in above three studies.

Meanwhile, recent studies support the correlation between *L. platyphylla* and glucose regulation, although there is no evidence for the role of glucose in the laxative effects of *L. platyphylla*. The homoisoflavone-enriched fraction and LP9M80-H from *L. platyphylla* increases the insulin-stimulated glucose uptake in 3 T3-L1 adipocytes and enhances the secretion of insulin in the HIT-T15 pancreatic ß-cell line [28, 29]. Also, LP9M80-H and aqueous extracts induce a significant increase of glucose levels and decrease in insulin concentrations in the blood of ICR mice and OLETF rats [29, 30]. Furthermore, similar effects on the insulin secretion by *L. platyphylla* have also been observed after EtRLP treatment; insulin secretion significantly increases after EtRLP treatment in INS cells, streptozotocin-induced diabetic ICR mice, and OLETF rats [13, 14, 31]. In the current study, we investigated the profile of endogenous metabolites in serum of constipation SD rats treated with EtRLP, to characterize putative biomarkers for laxative effects. We observed that the concentration of D-glucose dramatically recovers in the Lop + EtRLP treated group, as compared to the Lop + Veh treated group (Fig. 4). Therefore, we construe that the increased glucose concentration in the profile of endogenous metabolites may be caused by the ability of EtRLP for insulin secretion, although additional research is required to confirm the same.

Only a few studies have provided some evidences for the correlation between some metabolites and laxative effects. L-arginine effectively regulated the opioid-induced constipation via a stereospecific and peripheral action [32]. Average intake of taurine was significantly higher in functional constipation patients than control group [33]. Also, prucalopride succinate improved constipation through the stimulation of colonic motility [34]. Therefore, above results from previous studies support the reliability of metabolites identified by present study although additional studies will be need.

## Conclusions

Overall, we believe that the present research is the first study to examine metabolic changes in SD rats treated with Lop + EtRLP, and to correlate changes in specific metabolites consistent with laxative effects of EtRLP. In addition, the results presented herein provide evidence that 7 amino acids and 8 metabolites are potential biomarkers for predicting the laxative effects induced by natural product remedies.

## Materials and methods

### Preparation and analysis of EtRLP

EtRLP was prepared using the methods described in previous studies [31]. Briefly, to manufacture RLP from *L. platyphylla* (LP) at nine different steaming frequencies, the specific process comprising two steps (200 g of dry roots steamed at 99 °C for 3 h and air-dried at 70 °C for 24 h) was carried out for different numbers of repetitions a total of 9 times. The RLP was then subjected to the appropriate extraction process, and the resultant EtRLP was collected. Furthermore, the composition and the concentration of active compounds in the EtRLP were analyzed by the methods suggested in previous studies [12, 31].

### Experimental design for animals

The animal protocol used in this study was reviewed and approved based on ethical procedures and scientific care by the Pusan National University-Institutional Animal Care and Use Committee (PNU-IACUC; Approval Number PNU-2012-0010). Adult SD rats were purchased from Samtacho BioKorea Co. (Osan, Korea) and handled in a accredited by the Korea Food and Drug Administration (FDA) (Accredited Unit Number-000231) and AAALAC International (Accredited Unit Number; 001525). All rats were provided with standard irradiated chow diet (Purina Mills, Seoungnam, Korea) *ad libitum* and were maintained in a specific pathogen-free state under a strict light cycle (lights on at 06:00 h and off at 18:00 h) at a temperature of 22 ± 2 °C and a relative humidity of 50 ± 10%.

Constipation was induced in SD rats by subcutaneous injection of Lop (4 mg/kg weight) in 0.9% sodium chloride twice a day for 3 days, whereas the non-constipation group was injected with 0.9% sodium chloride alone [11]. For the experiment, 8-week-old SD rats (*n* = 30) were assigned to either a non-constipation group (*n* = 12) or a constipation group (*n* = 18). The non-constipation group was further divided into a No treated group (*n* = 6) and an EtRLP treated group (n = 6). The No treated group was untreated during the experimental period, whereas the EtRLP treated group received 15 μl/g body weight of EtRLP (1,000 mg/kg weight) one time. The constipation group was further divided into a Lop + Veh treated group (n = 6), Lop + EtRLP treated group (n = 6) and Lop + BisaC treated group (n = 6). The Lop + Veh treated group received a consistent volume of water via gavage, whereas the other cotreatment groups were administered once, either 1,000 mg/kg body weight of EtRLP (Lop + EtRLP treated group) or 3.3 mg/kg body weight of BisaC (Lop + BisaC treated group) after the induction of constipation. BisaC majorly included bisacodyl, docusate sodium and sennoside calcium, and was purchased from Kolon Pharmaceuticals INC. (Gyenggido, Korea). At 24 h after the EtRLP, BisaC and Veh treatment, all animals were euthanized using CO_2_ gas, and tissue samples were acquired and stored in Eppendorf tubes at − 70 °C until assay.

### Measurement of stool parameters and urinary volume

All SD rats were bred in metabolic cages during the entire experimental period to avoid contamination. Stools and urine excreted from each SD rat were collected daily at 10:00 a.m. Stool weight and number was measured three times per sample using an electric balance and hand counter, whereas the water content was determined as the difference between the wet and dry weights of the stool, as described previously [11, 31].

### Histological analysis

Colons collected from SD rats of subset group were fixed with 10% formalin for 48 h, embedded in paraffin wax, and then sectioned into 4 μm thick slices that were subsequently subjected to hematoxylin and eosin staining (H&E, Sigma-Aldrich Co., St. Louis, MO, USA). Morphological features, including thickness of mucosa and muscle, were observed under light microscopy at 100x and 400x magnification (Leica Microsystems, Switzerland).

### Metabolomics analysis

Appropriate serum samples (300 μl) obtained from animals were placed in micro centrifuge tubes containing 300 μl D_2_O solutions with 4 mM TSP as a qualitative standard for the chemical shift scale. After vortexing, serum samples were analyzed by NMR spectrometry within 48 h. All spectra were determined using a Varian Unity Inova 600 MHz spectrometer operating at a temperature of 26 °C supported by Pusan National University (Busan, Korea). The one-dimensional NMR spectra were acquired with the following acquisition parameters: spectral width 24,038.5 Hz, 7.55 min acquisition time, and 128 nt. Additional conditions of a relaxation delay time of 1 s and saturation power of 4 were set to suppress a massive water peak. NMR spectra were reduced to data using the program Chenomx NMR Suite (ver 4.6, Chenomx Inc., Edmonton, Alberta, Canada). The spectral region of δ0.0–10.0, excluding the water peak (δ4.5–5.0), was segmented into regions of 0.04 ppm, which provided 250 integrated regions in each NMR spectrum. This binning process endowed each segment with integral values, giving an intensity distribution of the whole spectrum with 250 variables prior to pattern recognition analysis.

All data were converted from the NMR suite Professional software format into Microsoft Excel format (*.xls). One-dimensional NMR spectra data were imported into the SIMCA-P (version 12.0, Umetrics Inc., Kinnelon, NJ, USA) for multivariate statistical analysis to examine the intrinsic variation in the data set. These data were scaled using centered scaling prior to principal component analysis (PCA) and partial least square-discriminant analysis (PLS-DA). With the scaling process, we calculated the average value of each variable, and then subtracted it from the data. Score plots of PCA and PLS-DA were used to interpret the intrinsic variation of the data. Variable importance plots (VIP) were also utilized to select putative metabolites related to loperamide.

### Serum biochemical analysis

After fasting for 8 h, whole blood from each rats in all groups was collected from their abdominal veins and incubated for 30 min at room temperature in a serum separating tube (BD Container, Franklin Lakes, NJ, USA). Serum was then obtained by centrifugation at 1,500×g and analyzed for D-glucose and creatinine using an automatic biochemical analyzer (BS-120, Mindray, China). All assays were conducted in duplicate using fresh serum.

### Statistical analysis

Statistical significance was evaluated using the One-way Analysis of Variance (ANOVA) (SPSS for Windows, Release 10.10, Standard Version, Chicago, IL, USA), followed by Tukey’s post hoc t-test for multiple comparison. All values are expressed as the means ± SD, and a *p* value < 0.05 is considered statistically significant.

## Additional file

Additional file 1: **Table S1.** Concentration of 33 metabolites in serum (DOCX 19 kb)

## Data Availability

The analyzed datasets generated during the present study are available from the corresponding author on reasonable request.
