# Supplementary material for: Metabolomics approach to serum biomarker for laxative effects of red Liriope platyphylla in loperamide-induced constipation of SD rats
Source: Lab Anim Res. 2019 Jul 24;35:9. doi: 10.1186/s42826-019-0009-x (PMC7081617; doi:10.1186/s42826-019-0009-x)
Supplement: Supplementary file 1 — Table S1. Concentration of 33 metabolites in serum (DOCX 19 kb) [file 42826_2019_9_MOESM1_ESM.docx]

Additional file 1

Table S1. Concentration of 33 metabolites in serum

| Parameters | No | EtRLP | Lop+Vehicle | Lop+EtRLP | Lop+BisaC |
| --- | --- | --- | --- | --- | --- |
| 2-Hdroxy isobutyrate | 2.26±1.04 | 3.33±1.56 | 2.70±0.21 | 10.01±11.31 | 8.47±10.96 |
| 3-Hydroxy butyrate | 1.36±1.61 | 1.18±1.09 | 0.64±0.40 | 3.56±4.46 | 3.13±4.48 |
| Acetate | 2.55±1.89 | 2.06±0.66 | 1.48±0.29 | 3.35±1.39 | 6.05±7.97 |
| Acetone | 0.58±0.74 | 0.31±0.20 | 0.17±0.05 | 0.86±1.02 | 0.5±0.59 |
| Alanine | 4.98±6.25 | 3.19±2.88 | 1.42±0.14 | 10.09±12.15 | 10.40±15.26 |
| Arginine | 2.27±2.78 | 1.48±1.57 | 0.79±0.10 | 5.93±7.81 | 4.43±6.58 |
| Betaine | 1.85±2.18 | 1.42±1.29 | 0.64±0.16 | 5.48±6.72 | 5.45±8.58 |
| Choline | 0.35±0.19 | 0.31±0.11 | 0.23±0.02 | 0.70±0.61 | 0.72±0.82 |
| Citrate | 1.02±0.66 | 1.24±0.97 | 0.59±0.13 | 2.81±3.31 | 2.39±3.64 |
| Creatine | 1.30±1.20 | 0.82±0.54 | 0.51±0.02 | 4.42±5.55 | 3.19±4.51 |
| Ethanol | 4.26±5.95 | 4.18±5.23 | 1.53±1.55 | 10.51±8.74 | 12.39±18.73 |
| Formate | 0.41±0.13 | 0.41±0.11 | 0.40±0.04 | 1.20±0.76 | 0.95±1.05 |
| Glucose | 175.2±243.7 | 85.07±95.46 | 25.11±6.03 | 247.6±298.2 | 212.4±314.2 |
| Glutamate | 3.36±4.16 | 3.62±3.86 | 1.12±0.22 | 8.26±8.91 | 7.50±10.90 |
| Glutamine | 5.66±7.36 | 4.48±4.92 | 1.28±0.09 | 12.44±15.39 | 10.31±15.76 |
| Glycerol | 3.55±4.02 | 3.02±2.18 | 1.24±0.12 | 6.98±8.23 | 5.93±8.37 |
| Glycine | 1.88±2.21 | 1.56±1.49 | 0.80±0.25 | 4.74±5.37 | 4.55±6.71 |
| Histidine | 0.42±0.48 | 0.46±0.42 | 0.18±0.06 | 1.06±1.31 | 0.93±1.37 |
| Isoleucine | 1.46±1.89 | 0.93±0.85 | 0.39±0.07 | 2.97±3.23 | 2.59±3.80 |
| Lactate | 38.54±47.50 | 41.96±43.62 | 16.12±3.57 | 142.8±182.4 | 115.3±175.7 |
| Leucine | 1.19±1.24 | 1.57±1.73 | 0.51±0.12 | 4.60±5.97 | 3.60±5.23 |
| Lysine | 1.43±1.78 | 0.82±0.50 | 0.50±0.08 | 0.67±0.21 | 0.51±0.25 |
| Malonate | 0.46±0.41 | 0.27±0.16 | 0.20±0.07 | 1.19±0.97 | 0.58±0.66 |
| Methionine | 0.73±0.85 | 0.49±0.38 | 0.26±0.03 | 1.46±1.50 | 0.97±1.36 |
| Phenylalanine | 0.64±0.77 | 0.44±0.31 | 0.25±0.07 | 1.09±1.06 | 1.01±1.35 |
| Pyruvate | 0.97±1.48 | 0.58±0.74 | 0.12±0.01 | 2.43±3.67 | 1.33±2.17 |
| Serine | 1.90±1.92 | 1.60±1.19 | 0.91±0.07 | 3.22±2.17 | 3.90±5.55 |
| Succinate | 5.62±6.38 | 4.70±3.74 | 2.21±0.36 | 14.64±17.02 | 16.10±24.04 |
| Taurine | 9.35±11.48 | 5.27±5.32 | 1.95±0.18 | 19.31±24.16 | 13.81±19.66 |
| Threonine | 3.03±4.35 | 1.94±1.85 | 0.79±0.11 | 4.77±4.49 | 5.17±7.64 |
| Trimethylamine | 0.06±0.05 | 0.07±0.06 | 0.03±0.01 | 0.35±0.39 | 0.23±0.38 |
| Tyrosine | 0.78±0.86 | 0.54±0.36 | 0.27±0.04 | 1.66±1.92 | 1.31±1.80 |
| Valine | 2.34±3.07 | 1.57±1.42 | 0.69±0.11 | 5.67±6.84 | 4.19±5.91 |

The data are reported as the mean ± SD.
